# Genome-wide identification of antisense lncRNAs and their association with susceptibility to *Flavobacterium psychrophilum* in rainbow trout

**DOI:** 10.3389/fimmu.2022.1050722

**Published:** 2022-12-06

**Authors:** Ali Ali, Mohamed Salem

**Affiliations:** Department of Animal and Avian Sciences, University of Maryland, College Park, MD, United States

**Keywords:** rainbow trout, *Flavobacterium psychrophilum*, bacterial cold water disease, lncRNA, antisense transcripts, immune genes, iron

## Abstract

Eukaryotic genomes encode long noncoding natural antisense transcripts (lncNATs) that have been increasingly recognized as regulatory members of gene expression. Recently, we identified a few antisense transcripts correlating in expression with immune-related genes. However, a systematic genome-wide analysis of lncNATs in rainbow trout is lacking. This study used 134 RNA-Seq datasets from five different projects to identify antisense transcripts. A total of 13,503 lncNATs were identified genome-wide. About 75% of lncNATs showed multiple exons compared to 36.5% of the intergenic lncRNAs. RNA-Seq datasets from resistant, control, and susceptible rainbow trout genetic lines with significant differences in survival rate following *Flavobacterium psychrophilum* (*Fp*) infection were analyzed to investigate the potential role of the lncNATs during infection. Twenty-four pairwise comparisons between the different genetic lines, infectious status, and time points revealed 581 differentially expressed (DE) lncNATs and 179 differentially used exons (DUEs). Most of the DE lncNATs strongly and positively correlated in expression with their corresponding sense transcripts across 24 RNA-Seq datasets. LncNATs complementary to genes related to immunity, muscle contraction, proteolysis, and iron/heme metabolism were DE following infection. LncNATs complementary to hemolysis-related genes were DE in the resistant fish compared to susceptible fish on day 5 post-infection, suggesting enhanced clearance of free hemoglobin (Hb) and heme and increased erythropoiesis. LncNATs complementary to hepcidin, a master negative regulator of the plasma iron concentration, were the most downregulated lncNATs on day 5 of bacterial infection in the resistant fish. Ninety-four DE lncNAT, including five complementary to hepcidin, are located within 26 QTL regions previously identified in association with bacterial cold water disease (BCWD) in rainbow trout. Collectively, lncNATs are involved in the molecular architecture of fish immunity and should be further investigated for potential applications in genomic selection and genetic manipulation in aquaculture.

## Introduction

Rainbow trout is one of the most important fish, but genome annotation is still incomplete compared to the model species such as zebrafish (1). For aquaculture breeding, a well-annotated reference genome sequence is essential for genomic-based animal selection (2). The human ENCODE project showed that the major category of the active genome transcription comprises noncoding RNAs (3). Noncoding RNAs can be distinguished into long and small noncoding RNAs based on their sizes. About 40% of long noncoding RNAs (lncRNAs) belong to the category of antisense transcripts, making them a substantial category of the noncoding RNA pool (4). Natural antisense transcripts (NATs) originate from the genomic regions opposite to coding or noncoding genes or introns of coding genes (5). Antisense transcription was previously identified in prokaryotes and eukaryotes (6–9). Previous studies identified a considerable antisense transcription from eukaryotic genomes of Arabidopsis (7.4%) (10, 11), fruit fly (16.8%) (12), zebrafish (49.3%) (13), mouse (72%) (14), and human (~61-72%) (14, 15).

Antisense transcripts regulate the function of the complementary protein-coding loci in *cis*-regulatory mechanisms (16, 17). Although antisense transcripts may also work in *trans* (4), *cis*-mechanisms are predominant because of NAT’s physical proximity to the overlapping loci (4, 18, 19). Antisense transcripts can either activate or repress the function of the sense protein-coding genes *via* a wide range of mechanisms (20). At the transcriptional level, antisense transcripts can induce promoter methylation (21, 22), interfere with the transcriptional machinery (23, 24), or recruit histone-modifying enzymes (25, 26). At the post-transcriptional level, antisense transcripts can regulate the splicing of sense protein-coding genes (27, 28) or interact with the sense mRNA and mask the miRNA binding sites to increase the mRNA stability (29). Furthermore, at the translational level, antisense transcripts can enhance mRNA translation by recruiting additional factors or degrade mRNA by creating endogenous siRNA (4, 30–32).

Recent evidence suggests that antisense transcripts have specific immune gene regulatory functions (33). For example, the expression of the C-C motif chemokine receptors 2, 3, and 5 and trafficking of Th2 cells to the lung are regulated by the antisense lincR-Ccr2-5’AS (34). Additionally, the antisense interleukin 1α (AS-IL1α) and its partially overlapping IL-1α protein-coding gene showed a correlation in expression. Loss of function short hairpin RNA (shRNA) approaches revealed that AS-IL1α facilitates IL-1α gene transcription (20). Conversely, the overexpression of the antisense transcript of interleukin 1β (AS-IL1β) decreases the histone modification (H3K4me3) located at the IL-1β promoter leading to decreased occupancy of RNA polymerase II and diminished IL-1β transcription, accordingly (35). Thus, identifying and characterizing immune-relevant antisense transcripts in the rainbow trout genome may help understand several diseases at the molecular level.

*Flavobacterium psychrophilum*, the causal agent of bacterial cold water disease (BCWD), causes worldwide economic losses to salmonid aquaculture (36). Rainbow trout mortality from BCWD varied worldwide with the highest mortality rate reaching 90% (37, 38). There is no effective commercial vaccine available for BCWD, in addition to limited chemotherapeutics and antibiotic resistance, making it difficult to prevent disease progression (39, 40). Selective breeding programs have potential to improve heritable phenotypes through existing genetic variation among individual animals and families (41). A family-based selection program was initiated in 2005 at the National Center for Cool and Cold Water Aquaculture (NCCCWA) by developing three rainbow trout genetic lines of variable BCWD resistance (42, 43). Studying the host-pathogen interactions will help developing new treatment and prevention strategies. Previous transcriptome profiling studies revealed transcriptional variation among the three fish genetic lines (control (ARS-Fp-C), resistant (ARS-Fp-R), and susceptible (ARS-Fp-S)) following *in vivo F. psychrophilum* (CSF259-93) infection (44, 45). A few lncNATs overlapping and exhibiting expression correlation with immune genes were differentially expressed (DE) among PBS- and Fp-injected fish of different genetic lines (45), perhaps because only 1,136 antisense transcripts were annotated in the reference (46). Thus, comprehensive genome-wide identification of lncNATs in rainbow trout is warranted. The objectives of this study were to 1) identify and characterize lncNATs in the rainbow trout genome, and 2) investigate the functional potential of lncNATs in the three genetic lines of rainbow trout in response to *Fp* infection and identify the potential biomarkers of BCWD infection and disease resistance. The current study provides a comprehensive genome-wide analysis of rainbow trout lncNATs and provides a resource for future genetic and genomic studies.

## Materials and methods

### Identification of lncNATs across the rainbow trout genome

A total of 134 public strand-specific RNA-Seq datasets were downloaded from Sequence Read Archive in GenBank. The data quality was checked as in our previous publications (1, 3). Trimmomatic v0.36 (47) was used, using default parameters, to remove the adaptor sequences and trim sequencing reads, followed by FastQC v0.11.8 (48) quality control checks. Trimmed reads used for downstream analyses had a quality score of Q30 or higher. Reads were mapped to the Oncorhynchus mykiss reference genome (GCF_002163495.1_Omyk_1.0) and then assembled into transcripts using HISAT2 2.1.0 (49) and StringTie v1.3.3 (50, 51), respectively, using default parameters. The assembled transcripts were inputted to Evolinc-I v1.5.1 (52) to identify trout’s long noncoding RNAs. In brief, transcripts less than 200 NT in length, those with an open reading frame (ORF) larger than 100 amino acids, and transcripts showing similarity to protein-coding genes with an E-value less than 10^-5^ were filtered out. Further, the remaining transcripts were categorized based on the transfrag class codes into *long intergenic RNAs* (lincRNAs) (class code U), sense overlapping transcripts (class code O), and lncNATs (class codes X and S). Transcripts were checked for predicted protein-coding potential (CPC (v1.0) score > -1 and CPAT (v1.2.3) score more than 0.35) (53, 54) or similarity to protein-coding domains in Pfam (55). In addition, BlastN (56) was used to filter out transcripts that have any match with other RNA families. LncNATs were located on the newly released Swanson (GCA_025558465.1) and Arlee (GCF_013265735.2) genome sequences by using minimap2-2.17 (r941) (57). SAM files were converted to gff files using a python script.

### Conservation analysis of lncNATs and lncNAT promoters

Genome FASTA sequences and GTF files of eight species (*Caenorhabditis elegans* (WBcel235 “GCA_000002985.3”), *Drosophila melanogaster* (BDGP6.32 “GCA_000001215.4”), *Danio rerio* (GRCz11 “GCA_000002035.4”), *Salmo salar* (Ssal_v3.1 “GCA_905237065.2”), *Anolis carolinensis* (AnoCar2.0v2 “GCA_000090745.2”), *Gallus gallus* (bGalGal1.mat.broiler.GRCg7b “GCA_016699485.1”), *Rattus norvegicus* (mRatBN7.2 “GCA_015227675.2”), and *Mus musculus* (GRCm39 “GCA_000001635.9”)) were downloaded from the Ensembl genome browser while rainbow trout genome (GCF_002163495.1_Omyk_1.0) and annotation file were downloaded from GenBank. Trout lncNATs and 500 NT upstream promoter sequences were used as inquiry sequences provided to FASTA36 (58). The latter was used to perform a series of BLASTn against the target sequences extracted from the Ensembl genomes of interest (E-value cutoff of 1E-20). The top blast hit with the lowest E-value and highest query coverage was identified for each genome and used for the downstream analysis.

### Fish population

To provide insights into the lncNAT biological roles, we downloaded RNA-Seq datasets generated by Marancik et al. (44) from three rainbow trout genetic lines ARS-Fp-R, ARS-Fp-C, and ARS-Fp-S experienced artificial selection based on BCWD post-infection survival. The challenge experiment was performed in the NCCCWA challenge facility with CSF-259-93 strain, as previously reported by Marancik et al. (44). In brief, fifty rainbow trout free of viral and bacterial pathogens were selected randomly from each genetic line and allocated to four tanks (4 tanks x 3 genetic lines x 50 fish/tank = 600 fish total). The mean body weights of the control, resistant, and susceptible fish were 1.12 ± 0.03 g, 1.11 ± 0.05 g, and 0.98 ± 0.04 g, respectively. For each genetic line, two tanks had *Fp*-injected fish (4.2 × 10^6^ CFU fish^-1^ *Fp* suspended in 10 μl of chilled PBS) and two tanks had PBS-injected fish (10 μl of chilled PBS alone). Injections were performed intraperitoneally using a repeater pipette fitted with a 27G × 1/2 inch needle. Five fish were collected from each tank on days 1 and 5 post-injection for RNA extraction. Fish survival was monitored for 21 days following injection. Whole body bacterial load in a subset of fish from the three genetic lines was measured by qPCR and expressed in terms of *Fp* genome equivalents (GE).

### RNA extraction, library preparation, and sequencing

RNA-Seq reads were downloaded from the Sequence Read Archive (accession number SRP047070). RNA extraction, library preparation, and sequencing were carried out as previously described by Marancik and coworkers (44). In brief, total RNA was extracted using TRIzol (Invitrogen, Carlsbad, CA) from whole fish with equal amounts pooled from five fish/tank at the two-time points (i.e., a total of 24 pools, n = 120 fish). Illumina’s TruSeq Stranded mRNA Sample Prep kit was used to prepare cDNA libraries following the manufacturer’s instructions. Three groups of eight indexed and barcoded libraries were sequenced in three lanes of an Illumina HiSeq 2000 (single-end sequencing, 100 bp read) at the University of Illinois at Urbana-Champaign.

### Identification of DE lncNATs and DUEs among fish genetic lines and co-expression analysis

Sequencing reads from 24 RNA-Seq datasets were mapped to the lncNATs using the CLC genomics workbench 9.5 (59). Raw counts were provided to DESeq2 v1.36.0 (60) and edgeR v3.14.0 (61) to identify DE lncNATs using default parameters. The output of DESeq2 and edgeR was further filtered based on log_2_ fold change and corrected *p*-values. The lncNAT was considered a significant DE if it had a log_2_ fold change larger than or equal to |1| at a *Padj* value less than 0.05. Expression correlation between lncNATs and complementary protein-coding genes was performed using the ExpressionCorrelation plugin (Version 1.1.0) in Cytoscape (62). DUEs (log_2_ fold change ≥ |1| and FDR < 0.05) were identified using DEXSeq Version 1.28.1 as previously described (1).

### Functional annotation of protein-coding genes opposite to lncNATs

For functional annotation, Gene Ontology (GO) terms were retrieved by uploading the complementary protein-coding gene sequences to lncNATs of interest to the eggNOG-mapper v2 (63, 64). GO terms were then uploaded to the CateGOrizer server (version 3.218) (65) to classify them in terms of the GO classes they belong to. In addition, a basic alignment search against the KEGG database through the KAAS-KEGG server Ver. 2.1 (66) was performed as previously described (67).

### Computational prediction of lncNAT targets

For RNA : RNA interactions, DE lncNATs, and their complementary protein-coding genes were used as an input to a locally installed LncTar program (Version 1.0) (68). The protein-coding genes used for this analysis encompassed the coding region and 3’UTR. The normalized deltaG (ndG) cutoff was set at -0.10. The 3’UTR interacting with lncNATs were screened for microRNA binding sites by using miRanda available in the sRNAtoolbox server (69) and RNA22 v2 (70) as previously described (3).

### Red blood cell count

Blood samples used in this study were obtained from the USDA/NCCCWA (Dr. Gregory D. Wiens). Blood was collected from non-infected fish produced from the resistant and susceptible genetic lines. Red blood cells were counted manually using the Neubauer hemocytometer as previously described (71).

## Results and discussion

### Genome-wide identification and characterization of lncNATs in rainbow trout

To identify lncNATs in rainbow trout, we downloaded 134 public strand-specific RNA-Seq datasets from five projects with the following accession numbers; SRP047070, SRP098572, SRP102416, SRP108797, and SRP131630. The RNA-Seq datasets were generated from 12 different tissues, fertilized eggs, and whole-body transcriptomes. For read mapping, we prioritized the Swanson genome because it has been widely used to identify genetic markers associated with complex traits, including disease resistance targeted in this study. The newest assembly of the Swanson line was released in 2022 but is not yet annotated by NCBI. So, the most recent annotated genome assembly available for the Swanson line (Omyk_1.0) (72) was used in this study. In addition, Vallejo et al. (73) mapped the recent and previous QTLs identified in association with BCWD, targeted in this study, to the latest annotated genome version of the Swanson line (Omyk_1.0). Thus, using the same reference genome (Omyk_1.0) in our study facilitated the accurate identification of DE lncNATs that overlap with previously identified BCWD QTL as reported in a subsequent section.

**Figure 1:**
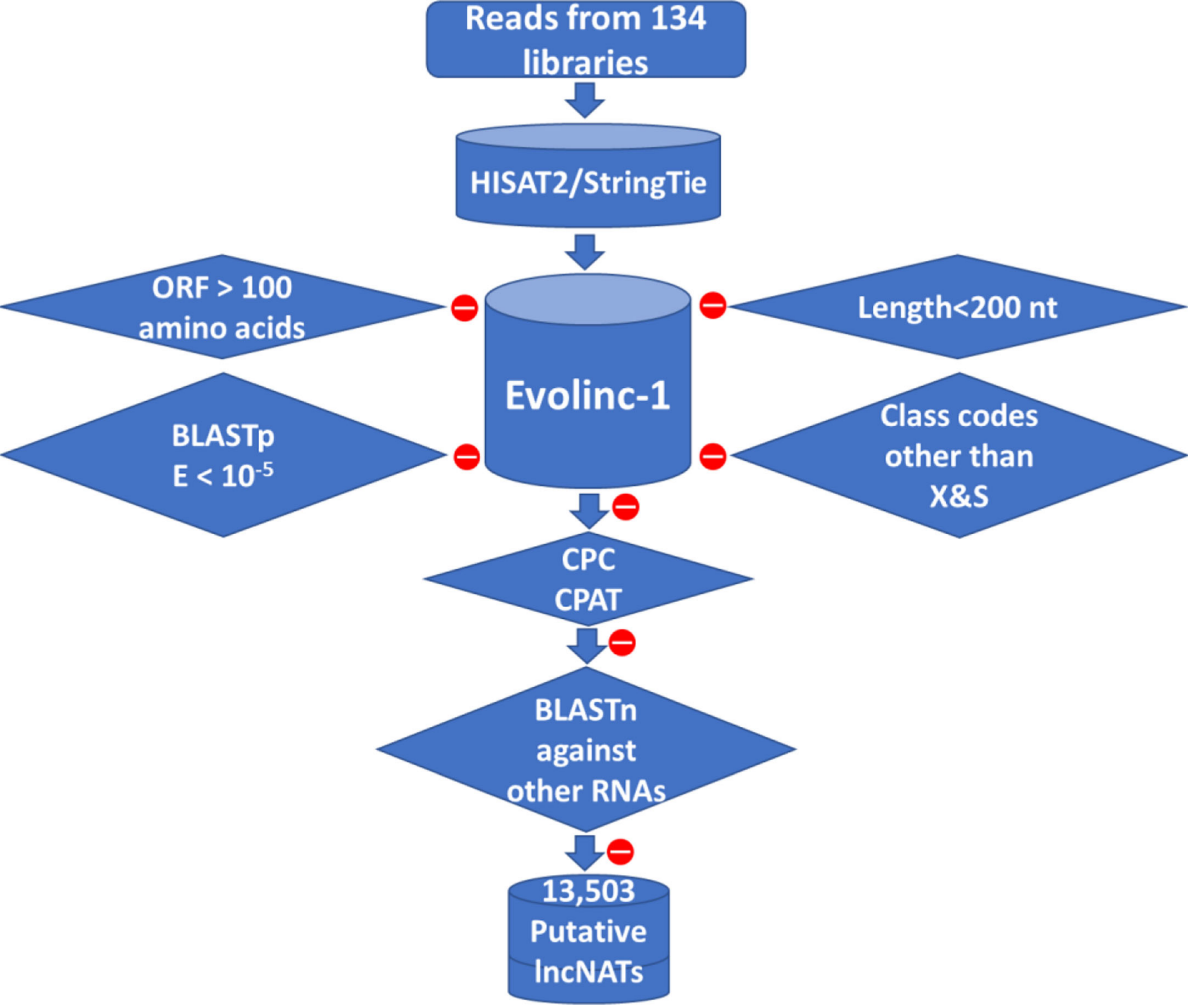
Bioinformatics pipeline used to predict lncNAT transcripts in rainbow trout. LncNATs were identified from 134 RNA-Seq datasets according to their length, coding potential, genomic location, and orientation relative to known genes. A total of 13,503 lncNAT transcripts were identified from all datasets. Filtration criteria applied to the assembled transcripts are represented in diamond shapes. Red circles mean transcripts were removed from the assembled pool of transcripts based on the shown filtration criterion.

To obtain a comprehensive lncNAT reference, mapped reads from each dataset were assembled into transcripts. A total of 469,678 transcripts from 208,940 genomic loci were assembled from all datasets. We used Evolinc-I (52) to identify trout lncNATs according to the bioinformatics workflow shown in **Figure 1** and as previously described (74). A total of 394,523 assembled transcripts were filtered out by Evolinc-I, yielding 57,375 potential lincRNAs (class code U), 3,755 sense overlapping transcripts (class code O), and 14,025 lncNATs (class codes X & S). Transcripts were checked for predicted protein-coding potential (CPC score > -1 and CPAT score > 0.35) or similarity to protein-coding domains in Pfam 32.0. In addition, Blastn was used to filter out transcripts with sequence matches to other RNA families such as tRNAs, rRNAs, and microRNA precursors. A total of 13,503 putative lncNATs passed all filters and were used for downstream analyses. Similar to the lncNATs, lincRNAs were subjected to a series of filtrations which yielded 56,527 potential lincRNAs to be used for comparative purposes with the lncNATs and mRNAs. Genomic coordinates of all lncNATs and lincRNAs are provided in **Supplementary File 1**. To compare the various genome assemblies, we mapped all the lncNATs identified in this study to the Arlee (Omyk_1.1) (75) and recent non-annotated Swanson (Omyk_2.0) genome sequences. Out of 13,503 transcripts, only 13 (0.09%) were not mapped to the Arlee genome. Also, when the lncNATs were mapped to the newly assembled non-annotated Swanson genome (Omyk_2.0), only 0.7% of the transcripts were not mappable. To facilitate future research, the genomic coordinates of lncNATs on the two recent Swanson (Omyk_2.0) and Arlee (Omyk_1.1) genomes were also provided in **Supplementary File 1**, which allows further investigation of lncNATs in future studies using the recent Arlee and Swanson genomes.

**Figure 2:**
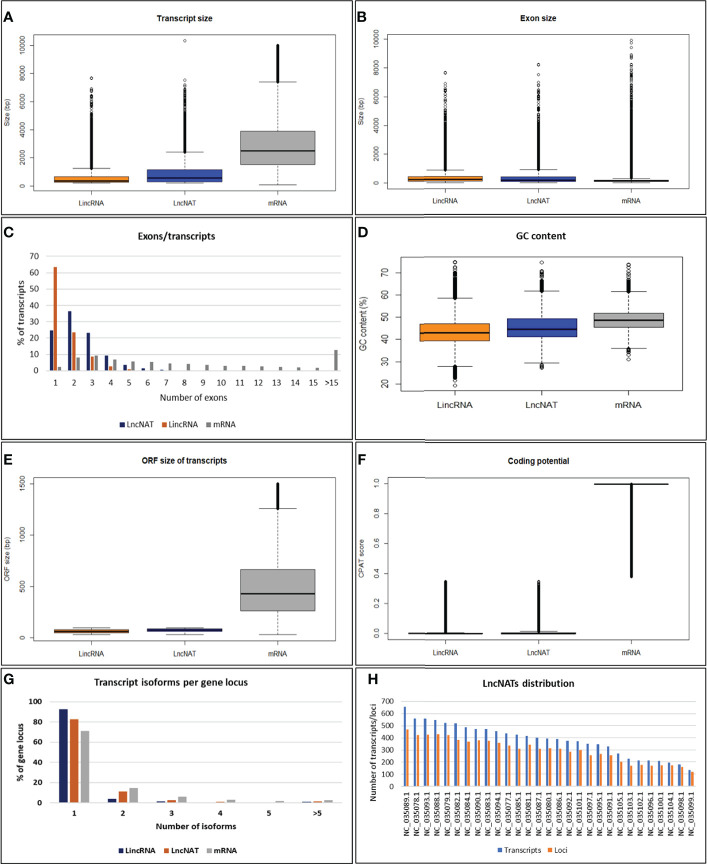
Genomic features of lncNATs compared to lincRNAs and mRNAs in rainbow trout. **(A)** The transcript size of lncNATs and lincRNAs is shorter than that of mRNAs. **(B)** The exon size of lncNATs and lincRNAs is longer than that of mRNAs. **(C)** LncNATs tend to have more exons than lincRNAs. **(D)** LncNATs have lower GC content than coding sequences but higher than lincRNAs. **(E, F)** LncNATs and lincRNAs have shorter ORF and lower coding scores than mRNAs. **(G)** LncNATs have fewer transcript isoforms per locus than coding sequences but more than lincRNAs. **(H)** Genomic distribution of lncNATs.

To characterize the genomic features of lncNATs, the putative lncNATs were compared to lincRNAs and protein-coding genes. The average length of LncNATs (908.19 bp) was longer than that of lincRNAs transcripts (578.66 bp) but significantly shorter than protein-coding genes (3111.30 bp, Kolmogorov-Smirnov test (KS-test) *p*-value = 0) (**Figure 2A**). Previous studies showed that lincRNA transcripts are shorter than lncNATs and protein-coding transcripts (74, 76). However, the exon size of lncNATs showed a different pattern in this study. LncNATs and lincRNAs had almost the same exon size (median length 376.5 bp and 371.0 bp) longer than that of the protein-coding genes (median length 173.5 bp) (**Figure 2B**). Notably, 74.63% of lncNATs were multi-exonic compared to 36.53% and 74.71% of lincRNAs and protein-coding genes, respectively (**Figure 2C**). The average number of exons in lncNATs was 2.41 compared to 1.56 and 9.33 in lincRNAs and protein-coding mRNA transcripts, respectively. This may explain the long exon size of both lncNATs and lincRNAs relative to protein-coding genes. We speculate that the multiple short exons in the mRNA can facilitate the generation of multiple transcript isoforms from the same gene locus, maximizing the production of variant proteins. The GC content in trout lncNATs was lower than in coding sequences but higher than in lincRNAs (**Figure 2D**). The median size of the maximum ORF per lncNATs was 72 bp and 62 bp in lincRNAs, which was significantly shorter than that of mRNA (635 bp) (**Figure 2E**). CPAT Protein-coding potentials scores for lncNATs and lincRNAs averaged 0.02 compared to 0.97 in protein-coding genes (**Figure 2F**). The number of exons, GC content, the median size of ORF, and coding scores in all three types of transcripts showed a similar pattern to other species, suggesting evolutionarily conserved genome structures (13, 74, 76–80). A large fraction of the lncNATs (83.6%) had at least 50% of their sequence length overlapped with protein-coding genes. Of them, 36.6% were bi-exonic, and 25.4% were monoexonic. LncNAT genomic loci had a fewer number of transcript isoforms (1.4 per gene locus) than mRNA loci (1.7 per gene locus), suggesting less complexity of lncNATs compared to mRNAs as shown in other species (76) (**Figure 2G**). Trout lncNATs were unevenly distributed across the 29 chromosomes (**Figure 2H**). Chromosome 13 (NC_035089.1) had the largest number of lncNATs with the highest gene density (656 transcripts, 471 gene loci, 9.9 genes/Mb), whereas chromosome 23 (NC_035099.1) contained the least number and lowest density of lncNATs (135 transcripts, 119 gene loci, 2.8 genes/Mb). The chromosome size and lncNAT gene density were significantly correlated (R = 0.54, *p*-value = 0.003). About one-quarter of lncNATs (28.1%) showed a relatively high expression correlation with their cognate protein-coding genes across the 24 RNA-Seq datasets. Of note, 99.7% of the sense/antisense correlations were positive as consistently reported in other species (14, 81–85).

**Figure 3:**
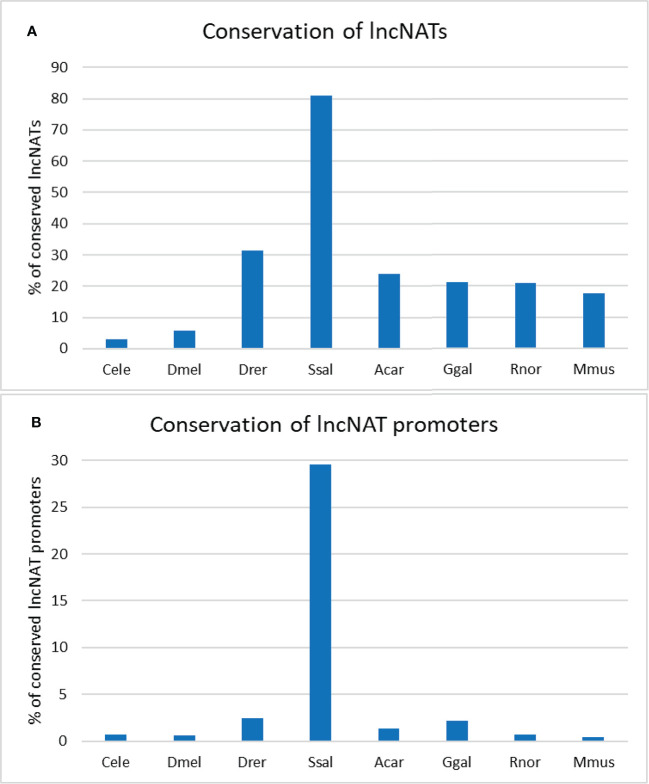
Percentage of conserved rainbow trout’s lncNATs and their promoters across closely- and distantly-related species. Trout’s lncNATs **(A)** and their promoters **(B)** are more conserved in Atlantic salmon and zebrafish. Each species is represented on the X-axis by the first letter of the genus name and the first three letters of the species name; (*Caenorhabditis elegans* “Cele”, *Drosophila melanogaster* “Dmel”, *Danio rerio* “Drer”, *Salmo salar* “Ssal”, *Anolis carolinensis* “Acar”, *Gallus gallus* “Ggal”, *Rattus norvegicus* “Rnor”, and *Mus musculus* “Mmus”).

Since sequence conservation may imply functionality (86), we sought to identify the conserved lncNATs. For this purpose, we searched for conserved transcripts across eight species (*Caenorhabditis elegans*, *Drosophila melanogaster*, *Danio rerio*, *Salmo salar*, *Anolis carolinensis*, *Gallus gallus*, *Rattus norvegicus*, and *Mus musculus*). The lncNATs showed a low level of sequence conservation in seven species. Almost 81% of lncNATs were conserved in *Salmo salar*, explained by the phylogenetic relationship with rainbow trout, followed by about 31.4.8% in *Danio rerio*. LncNATs showed less conservation in *Anolis carolinensis*, *Gallus gallus*, *Rattus norvegicus*, and *Mus musculus* than fish species. LncNATs had the lowest level of conservation with the distantly-related species; *Caenorhabditis elegans* (3.1%) and *Drosophila melanogaster* (5.7%) (**Figure 3A**). We identified 301 ultraconserved elements across all eight species and 1,943 lncNATs among the six vertebrate species (*Danio rerio*, *Salmo salar*, *Anolis carolinensis*, *Gallus gallus*, *Rattus norvegicus*, and *Mus musculus*). Analysis of lncNAT promoters revealed a lower level of conservation than lncNATs (*p*-value = 0.006) across the eight species (**Figure 3B**). In agreement with our findings, about 14%, 3.56%, 0.5%, and 39% of antisense noncoding transcripts exhibited limited conservation among closely related bacteria, insects, plants, and mammals, respectively (74, 87–89). In bacteria, antisense transcript promoters did not show evidence of sequence conservation (87). Although sequence conservation implies functionality (86), a lack of conservation does not imply a loss of functionality (86). Therefore, more effort is needed to investigate the function of lncNATs, especially the conserved ones, under different biological conditions.

### Global lncNATs expression in BCWD-resistant, -susceptible, and -control genetic lines of rainbow trout

**Figure 4:**
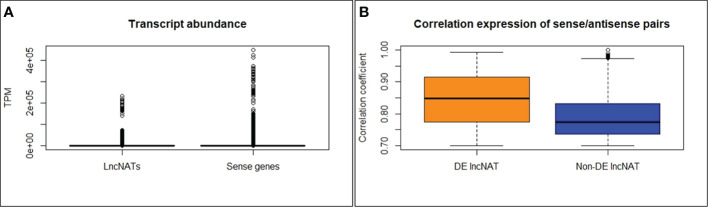
LncNATs are less abundant than their sense counterparts **(A)**. DE lncNATs exhibited a higher expression correlation with their sense protein-coding genes than non-DE lncNATs **(B)**.

The second objective of this study was to identify lncNATs associated with genetic resistance against BCWD and identify immune-related genes associated with lncNATs expression. For this purpose, we utilized sequencing data generated from three genetic lines (resistant (ARS-Fp-R), susceptible (ARS-Fp-S), and control (ARS-Fp-C)), generated by the NCCCWA following *F. psychrophilum* (CSF259-93) infection. mRNA and lncRNA expressions were previously analyzed in the three genetic lines after 1 and 5 days of the *Fp* challenge (44, 45). In the previous studies, 51.8% and 8.2% of the total RNA-Seq reads (518,881,838) were mapped to the mRNA and lncRNA references, respectively (44, 45). In the current study, 70,863,638 (13.68%) of the total RNA-Seq reads were mapped to the lncNATs reference suggesting that lncNAT is a substantial category of the fish transcriptome. A considerable antisense transcription was previously reported from eukaryotic genomes of Arabidopsis (7.4%) (10, 11), *fruit fly* (16.8%) (12), zebrafish (49.3%) (13), mouse (72%) (14), and human (~61-72%) (14, 15). A total of 12,092 lncNATs (89.55%) were expressed in all genetic lines at TPM ≥ 0.5 compared to 87.2% of lncRNAs. Of the 71,232 protein-coding transcripts, 9,095 (12.7%) had antisense transcription on the opposite strand. In humans, more than 30% of the annotated transcripts have counterpart antisense transcripts (18). The antisense transcripts were, on average, 2.6-fold lower in abundance than their sense counterparts’ expression across 24 RNA-Seq datasets of fish from different genetic lines and infectious conditions (**Figure 4A**).

Pairwise comparisons between the three genetic lines, infectious status, and time points of infection were performed using DESeq2 and identified 1,376 (581 non-redundant) DE lncNATs in all comparisons (Fold change ≥ |2|, FDR < 0.05). For confirmation, edgeR was also used for differential gene expression analysis and identified 4,802 (1,393 non-redundant) DE lncNATs in all comparisons. Remarkably, 554 of the non-redundant DE lncNATs (95.4%) identified by DESeq2 were also detected by the edgeR. Since DESeq2 was previously used to identify DE mRNAs (44), we used the DESeq2 output for all downstream analyses in the current study. A list of all DE lncNATs is provided in **Supplementary File 2**. DE lncNATs and DE protein-coding genes showed a higher correlation coefficient (R = 0.81, *p*-value = 1.72E-06; **Table 1**) than that of DE lncNATs and DE lincRNAs (R = 0. 6, *p*-value = 0.002). We previously reported a moderate correlation between DE protein-coding genes and DE lncRNAs (R = 0.63, *p*-value = 0.001) (45). For each pairwise comparison, there were fewer DE lncNATs than DE protein-coding genes except in the PBS-injected resistant and susceptible genetic lines relative to the control line on day 1 (**Table 1**). The number of DE lncNATs was greater than that of DE lncRNAs in 18 comparison groups. Challenging the fish with the pathogen increased the number of DE lncNATs in all different genetic lines from day 1 to day 5. We noticed an increase in the number of DE lncNATs in the infected ARS-Fp-S line on day 5 (138 lncNATs) compared to day 1 (71 lncNATs). On the other hand, the infected ARS-Fp-R line showed a slight increase in the numbers of DE lncNATs on day 5 (54 lncNATs) than on day 1 (50 lncNATs) (**Table 1**).

**Table 1:** Comparison of DE lncNATs, lncNAT DUEs, lncRNAs, and protein-coding genes in response to PBS/Fp injection..

Comparison	Day, genetic line, and infectious status	DE mRNA	DE lncRNA	DE lncNATs	DUEs
Infected vs. PBS	Day 1 R-line (Fp) vs. R-line (PBS)	515	57	50	8
	Day 5 R-line (Fp) vs. R-line (PBS)	428	36	54	22
	Day 1 C-line (Fp) vs. C-line (PBS)	20	0	13	5
	Day 5 C-line (Fp) vs. C-line (PBS)	2,201	54	62	8
	Day 1 S-line (Fp) vs. S-line (PBS)	1,663	125	71	9
	Day 5 S-line (Fp) vs. S-line (PBS)	2,225	196	138	12
Genetic lines (PBS)	Day 1 R-line (PBS) vs. S-line (PBS)	76	24	22	19
	Day 1 R-line (PBS) vs. C-line (PBS)	3	2	27	17
	Day 1 S-line (PBS) vs. C-line (PBS)	28	6	39	9
	Day 5 R-line (PBS) vs. S-line (PBS)	45	22	25	13
	Day 5 R-line (PBS) vs. C-line (PBS)	246	28	9	9
	Day 5 S-line (PBS) vs. C-line (PBS)	61	25	24	17
Genetic lines (*Fp*)	Day 1 R-line (Fp) vs. S-line (Fp)	150	15	55	2
	Day 5 R-line (Fp) vs. S-line (Fp)	1,016	83	103	20
	Day 1 R-line (Fp) vs. C-line (Fp)	28	12	26	7
	Day 5 R-line (Fp) vs. C-line (Fp)	159	21	45	36
	Day 1 S-line (Fp) vs. C-line (Fp)	37	13	17	10
	Day 5 S-line (Fp) vs. C-line (Fp)	1,758	5	114	31
Time points	Day 5 vs. Day 1 R-line (PBS)	1,286	26	80	10
	Day 5 vs. Day 1 C-line (PBS)	294	36	40	8
	Day 5 vs. Day 1 S-line (PBS)	376	14	66	14
	Day 5 vs. Day 1 R-line (Fp)	334	22	57	16
	Day 5 vs. Day 1 C-line (Fp)	2,469	70	169	17
	Day 5 vs. Day 1 S-line (Fp)	2,434	45	70	4

Four comparisons between Fp- vs. PBS-injected genetic lines, PBS-injected genetic lines, Fp-injected lines, and day 1 vs. day 5 injection. LncNAT was considered DE at l fold change ≥ |2| and Padj < 0.05.

Alternative splicing (AS) is an interesting aspect of the eukaryotic transcriptome to generate more transcript isoforms and increase the repertoire of proteins (90). Previous studies showed that AS plays a crucial role in immune response and diseases (1, 91). In particular, our previous study identified changes in the relative usage of protein-coding exons between BCWD-resistant and -susceptible fish. For instance, exon skipping was detected in a gene encoding interferon-induced very large GTPase 1 (GVIN1) in fish susceptible to BCWD (1). In this study, we sought to profile differential usage of NAT exons in the three genetic lines under different infectious statuses and time points of infection. Pairwise comparisons identified 323 (179 non-redundant) DUEs in all comparisons (Fold change ≥ |2|, FDR < 0.05) (**Table 1**). DUEs belong to 334 antisense transcripts encoded by 115 gene loci. Notably, DUEs-harboring lncNATs were not mainly DE at the transcript level. The complete list of all DUEs lncNATs is provided in **Supplementary File 2**.

### Relationship between DE lncNATs and their sense immune-related loci

LncNATs were classified as exonic or intronic according to their genomic location relative to their sense coding loci. We reported the classification of all 581 DE lncNATs in **Supplementary File 2**. To gain insights into the implications and biological roles of DE lncNATs in fish immune defense against infection, we annotated their sense coding loci and defined involvement in the signaling and immune pathways. Functional annotation of all sense protein-coding genes revealed that 31.77%, 14.94%, 7.92%, and 6.02% were involved in metabolism, stress response, response to external stimulus, and immune response suggesting a potential role of DE lncNATs in the fish immune response. Of the 429 protein-coding sense genes linked to DE lncNATs, 205 were successfully mapped and had hits to various KEGG pathways. Of them, 31 transcripts were involved in immune pathways (such as complement component 4 and interleukin 1 beta), and 40 transcripts were involved in signaling pathways (such as MAPK signaling pathway, NF-kappa B signaling pathway, and TNF signaling pathway), whereas 22 transcripts were mapped to both immune and signaling pathways. Common transcripts included interleukin 1 beta, C-C motif chemokine 13, chemokine CK-1 precursor, mast/stem cell growth factor receptor kita, TGF-beta receptor type-2, and T-cell receptor alpha chain C region. In addition to genes mapped to the KEGG immune and signaling pathways, the list of sense genes includes transcripts with multi-faceted roles, including immune response/inflammation. These transcripts include E-selectin, hepcidin, haptoglobin, HMG box-containing protein 1, and periostin.

To investigate the potential relationship between lncNATs and their sense loci, we compared their expression levels across 24 RNA-Seq datasets representing different genetic lines and infectious status. We used normalized TPM values to cluster the expressed transcripts. Most of the sense-lncNAT pairs showed a positive correlation in expression. Only 10 lncNATs showed a negative expression correlation with their cognate protein-coding loci. However, none of the 10 lncNATs were DE. The correlated DE lncNATs and their sense genes are listed in **Supplementary File 2**. In total, 252 DE lncNATs (43.4%) showed strong correlations with their sense protein-coding genes (R ≥ 0.70). DE lncNATs exhibited a higher expression correlation with their sense protein-coding genes than non-DE lncNATs (**Figure 4B**). Previous studies showed that sense-antisense pairs usually exhibit positive expression correlations (14, 81–85). Lysozyme II was among the immune-relevant genes with a positively correlated DE lncNAT (R = 0.90). Lysozymes hydrolyze the peptidoglycan backbone of the gram-negative bacteria and initiate the innate immune system to clear the infection (92). Similarly, liver-expressed antimicrobial peptide 2B showed a positive correlation with its DE lncNAT (R = 0.80). Liver-expressed antimicrobial peptide 2 was induced in response to *Salmonella enterica* infection in chicken and is a part of the innate immune system (93). In addition, 47 uncharacterized protein-coding transcripts were correlated in genomic location and expression with the counterpart DE lncNATs. These transcripts may have immune-related functions.

### LncNATs complementary to immune-related genes were regulated during the early and late response to infection

#### Early response profile on day 1

Herein, we investigated the pairwise comparisons between *Fp*-infected fish and PBS-injected fish from the same genetic line on day 1 (**Supplementary File 2**). For the ARS-Fp-S susceptible line, 72 lncNATs were DE in response to the Fp infection on day 1. Of them, 49 lncNATs were upregulated. The list included lncNATs overlapping with immune-related genes such as chemokine CK-1, Interleukin-8 (IL-8), E-selectin, mast/stem cell growth factor receptor kita, roquin-2, interferon-induced protein 44, and suppressor of cytokine signaling 3. Sense genes showed significant enrichment in GO terms linked to cellular homeostasis, positive regulation of leukocyte migration, response to external stimulus, leukocyte chemotaxis, and regulation of leukocyte migration. For instance, IL-8, also known as permeability factor 2, is a potent chemoattractant of neutrophils (PMN) and an activator of PMN transendothelial migration (94). On the other hand, 22 lncNATs were downregulated in susceptible fish following infection on day 1. Downregulated lncNATs overlapped with loci coding for immune-related genes such as C-C motif chemokine 13, small inducible cytokine A13, and engulfment and cell motility protein 2. Differential exon usage (DEU) analysis revealed that nine exonic regions were differentially used between Fp- and PBS-injected susceptible fish. Remarkably, five of them demonstrated exon skipping in antisense transcript isoforms overlapping with a gene encoding serum albumin 1 (**Supplementary File 2**). The latter has previously demonstrated a role in innate immunity by inhibiting the growth of pathogenic microorganisms (95).

**Figure 5:**
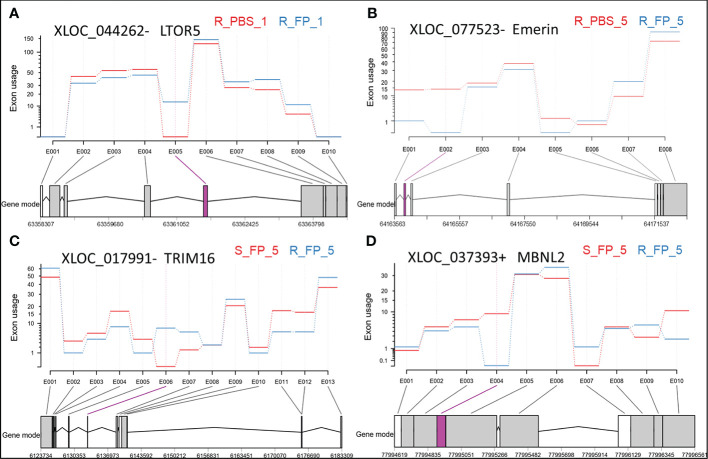
Exonic regions showing differential usage in antisense transcripts overlapping with LTOR5 **(A)**, Emerin **(B)**, TRIM16 **(C)**, and MBNL2 **(D)**. Significant differentially used exons (DUEs) are represented in pink at the bottom of each panel. Each biological condition is represented by the genetic line (R or S), type of injection (FP or PBS), and days post-injection (day 1 or day 5).

On the other hand, the ARS-Fp-R resistant fish line displayed less response relative to the ARS-Fp-S susceptible line. We identified 50 DE lncNATs; 38 lncNATs were upregulated in the infected fish. Similar to the susceptible genetic line, sense genes showed significant enrichment in GO terms linked to positive regulation of leukocyte migration, response to external stimulus, leukocyte chemotaxis, and regulation of leukocyte migration. Seventeen lncNATs showed a similar expression pattern as in the ARS-Fp-S line (upregulated). These lncNATs were overlapping mainly with immune-related genes such as E-selectin, chemokine CK-1 precursor, interferon-induced protein 44, and suppressor of cytokine signaling 3. DEU analysis revealed differential usage of eight exons between Fp- and PBS-injected resistant fish. Interestingly, exon inclusion in two lncNATs complementary to a gene encoding ragulator complex protein LAMTOR5 (LTOR5) was observed in Fp-injected resistant fish (**Figure 5A** and **Supplementary File 2**). LTOR5 is an autophagy inhibitory protein that acts as an activator of the potent autophagy inhibitor mTORC1 (96). Autophagy plays a crucial role in the immune response and defense against many microbial pathogens. Some pathogens disrupt autophagy to form a replicative niche (97). The DE lncNATs and DUEs involved in the early response are listed in **Supplementary File 2**.

#### Late response profile on day 5

To estimate the late response to infection, we investigated pairwise comparisons between the *Fp*-infected and PBS-injected fish on day 5 (**Supplementary File 2**). The ARS-Fp-S susceptible genetic fish line exhibited a higher number of DE transcripts (n = 138 lncNATs) than the ARS-Fp-R resistant line (n = 54 lncNATs). Of the 54 DE lncNATs, 18 were shared between the two genetic lines. A NAT complementary to differentially regulated trout protein 1 was the most upregulated common NAT during the late response in ARS-Fp-S and ARS-Fp-R genetic lines. High levels of differentially regulated trout protein 1 transcript indicate that the acute phase response has been activated (98). LncNATs complementary to other immune-related genes, including complement factor H, lysozyme II, complement C4, and interferon-induced protein 44, were also shared between the two genetic lines during late response. In Fp-infected resistant and susceptible fish, DEU analysis revealed two common exon-skipping events in lncNATs complementary to myosin and an uncharacterized transcript (**Supplementary File 2**).

The ARS-Fp-R genetic line had 36 unique DE lncNATs during the late response compared to the ARS-Fp-S line. For example, the ARS-Fp-R line had eight downregulated lncNATs complementary to loci coding for proteolytic enzymes, including trypsin, chymotrypsin, cathepsin L2, and carboxypeptidase B. In humans, the proteolytic MEP1A gene is a susceptibility gene for inflammatory bowel disease (99). Also, the resistant fish line had eight downregulated lncNATs overlapping with uncharacterized proteins, which warrants further investigation. Fish from the resistant genetic line also had 20 unique DUEs during the late response compared to the susceptible fish (**Supplementary File 2**). LncNATs complementary to genes encoding myosin heavy chain and emerin (**Figure 5B**) were at the top of those demonstrating exon skipping in resistant fish on day 5 post-infection. Genes encoding emerin have previously shown an association with Emery-Dreifuss muscular dystrophy (100).

**Figure 6:**
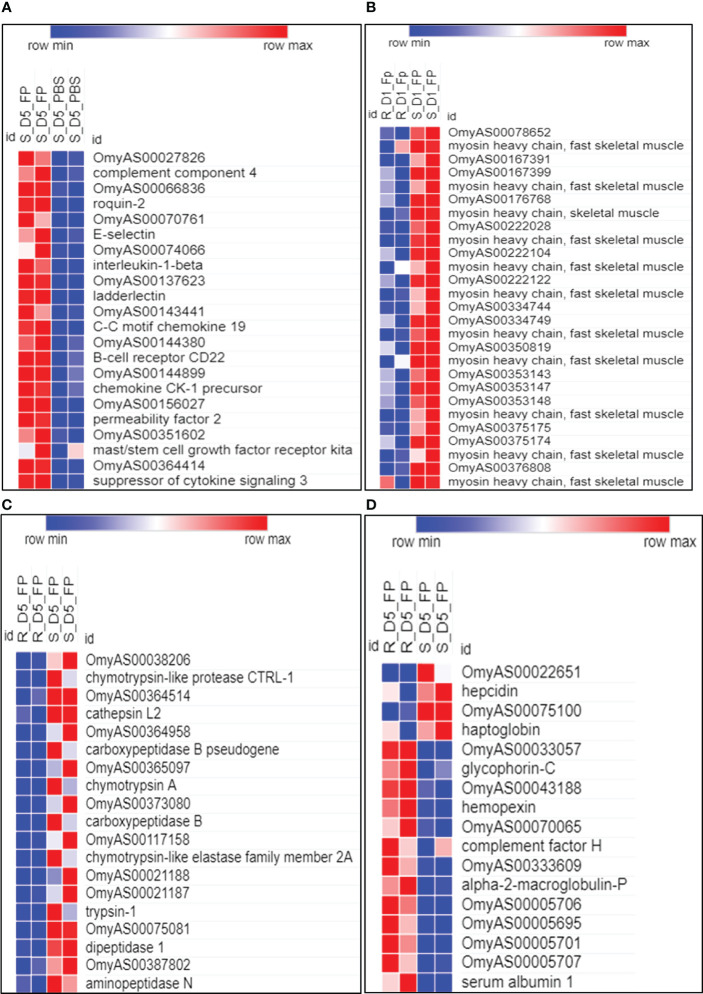
Comparison of transcriptome abundance of selected DE lncNATs and their sense genes. **(A)** Unique DE lncNATs, complementary to immune-related genes, in susceptible fish on day 5 post-infection. **(B–D)** DE lncNATs between resistant and susceptible genetic lines overlap with genes related to muscle contraction **(B)**, proteolysis **(C)**, and iron homeostasis **(D)**. Normalized expression from sense genes is visualized on the heatmap below the corresponding lncNAT(s). D1 and D5 indicate day 1 and day 5 post-challenge, respectively. FP and PBS indicate *Fp* and PBS injection, respectively. R and S represent resistant and susceptible genetic lines of the fish.

On the other hand, the ARS-Fp-S susceptible fish line had 120 unique DE lncNATs (73 upregulated) compared to the ARS-Fp-R line. Several of these lncNATs are complementary to loci coding for immune-related genes suggesting infection triggered inflammation/immune response due to the high bacterial load on day 5 (**Figure 6A**). Sense immune-related genes included permeability factor 2, chemokine CK-1 precursor (CXCR1), interleukin-1-beta (IL-1β), complement component 4, B-cell receptor CD22, mast/stem cell growth factor receptor kita, roquin-2, and C-C motif chemokine 19. By increasing vascular permeability, the permeability factor triggers the wound healing response (101). Also, the loss of human CXCR1 impaired the host defense against *Pseudomonas* (102). Functional enrichment analysis of the sense genes revealed GO terms linked to positive regulation of leukocyte migration, leukocyte chemotaxis, cellular homeostasis, positive regulation of immune system process, and response to external stimulus. IL-1β is a potent pro-inflammatory cytokine that protects against bacterial infections by activating several immune responses, such as the rapid recruitment of neutrophils to inflammatory sites (103). Fish from the susceptible genetic line had also 10 unique DUEs during the late response compared to the resistant fish (**Supplementary File 2**). A lncNAT complementary to a gene encoding myosin heavy chain was at the top of those demonstrating exon skipping in susceptible fish on day 5 post-infection. The list of DE lncNATs and DUEs during the late response and their expression correlations across 24 RNA-Seq datasets were provided in **Supplementary File 2**.

### LncNATs complementary to genes involved in muscle contraction, proteolysis, and heme/iron metabolism were regulated in the genetic lines following infection

We noticed a differential expression of lncNATs complementary to non-immune genes during the early and late response. For instance, we observed differential expression of lncNATs complementary to myosin heavy chain genes, proteolytic genes, and genes involved in cellular iron ion homeostasis (**Figures 6B–D**). To identify the contribution of these non-immune genes to disease resistance, we compared *Fp*-infected fish of ARS-Fp-S and ARS-Fp-R genetic lines at the same time points. Fifty-six lncNATs were DE between the susceptible and resistant genetic lines on day 1 following infection (**Supplementary File 2**). Seventeen lncNATs complementary to 12 myosin heavy chain loci were upregulated in susceptible fish compared to resistant on day 1 following *Fp*-infection (**Figure 6B**). All myosin transcripts and complementary lncNATs exhibited strong positive expression correlations across the 24 RNA-Seq datasets. Furthermore, exon usage analysis revealed differential usage of two exons (**Supplementary File 2**). A lncNAT overlapping with myosin was the top transcript showing exon skipping in resistant fish on day 1 post-infection. Inactivity or reduced swimming activity has been reported recently to be associated with disease resistance in salmon (104). In *Salmo salar*, myosin was upregulated in susceptible fish in response to sea lice infection (104), suggesting myosin loci and complementary lncNATs as potential markers for BCWD susceptibility.

On the other hand, we identified 103 DE lncNATs and 20 DUEs between ARS-Fp-R and ARS-Fp-S genetic lines on day 5 (**Supplementary File 2**). Most DUEs, which demonstrated exon inclusion in resistant fish, belong to antisense transcript isoforms overlapping with genes important for protein degradation and turnover, such as tripartite motif-containing protein 16 (TRIM16; **Figure 5C**) (105), cathepsin D (106), and ubiquitin-conjugating enzyme E2 L3 (107). Conversely, antisense transcripts overlapping with muscleblind-like protein 2 (MBNL2) were at the top of those showing exon skipping in Fp-infected resistant fish (**Figure 5D** and **Supplementary File 2**). MBNL2-deficient mice developed myotonia and skeletal myopathy, critical features of myotonic dystrophy (108). Our previous study showed that exon 3 in isoforms encoding dystrophin was completely absent in fish belonging to the resistant genetic line (1). Consistent with the hemolytic activity of *Flavobacterium* (109), the resistant fish had differentially expressed genes synchronized toward reducing hemolysis. For example, fish of the ARS-Fp-R genetic line had an upregulated NAT complementary to glycophorin-C, a red blood cell membrane protein that maintains the cell shape and regulates the membrane’s mechanical stability (110). In addition, the complement factor H and its complementary NAT were upregulated in the resistant fish. The deficiency of complement factor H leads to hemolytic diseases (111). Moreover, ten lncNATs and their complementary genes encoding proteinases/peptidases were upregulated in the susceptible fish (**Figure 6C**). The list of the proteinases/peptidases (n = 9) includes trypsin-1, chymotrypsin A, carboxypeptidase B, dipeptidase 1, and aminopeptidase N. Previous studies revealed that proteolytic enzymes, such as trypsin-1, can promote the hemolytic activity (112). We also identified four upregulated lncNATs complementary to the DE E-selectin in the susceptible fish line. Adhesion molecules, including E-selectin, expressed in the vascular endothelial cells, are induced by heme (113), suggesting a higher hemolysis rate in susceptible fish relative to the resistant.

**Figure 7:**
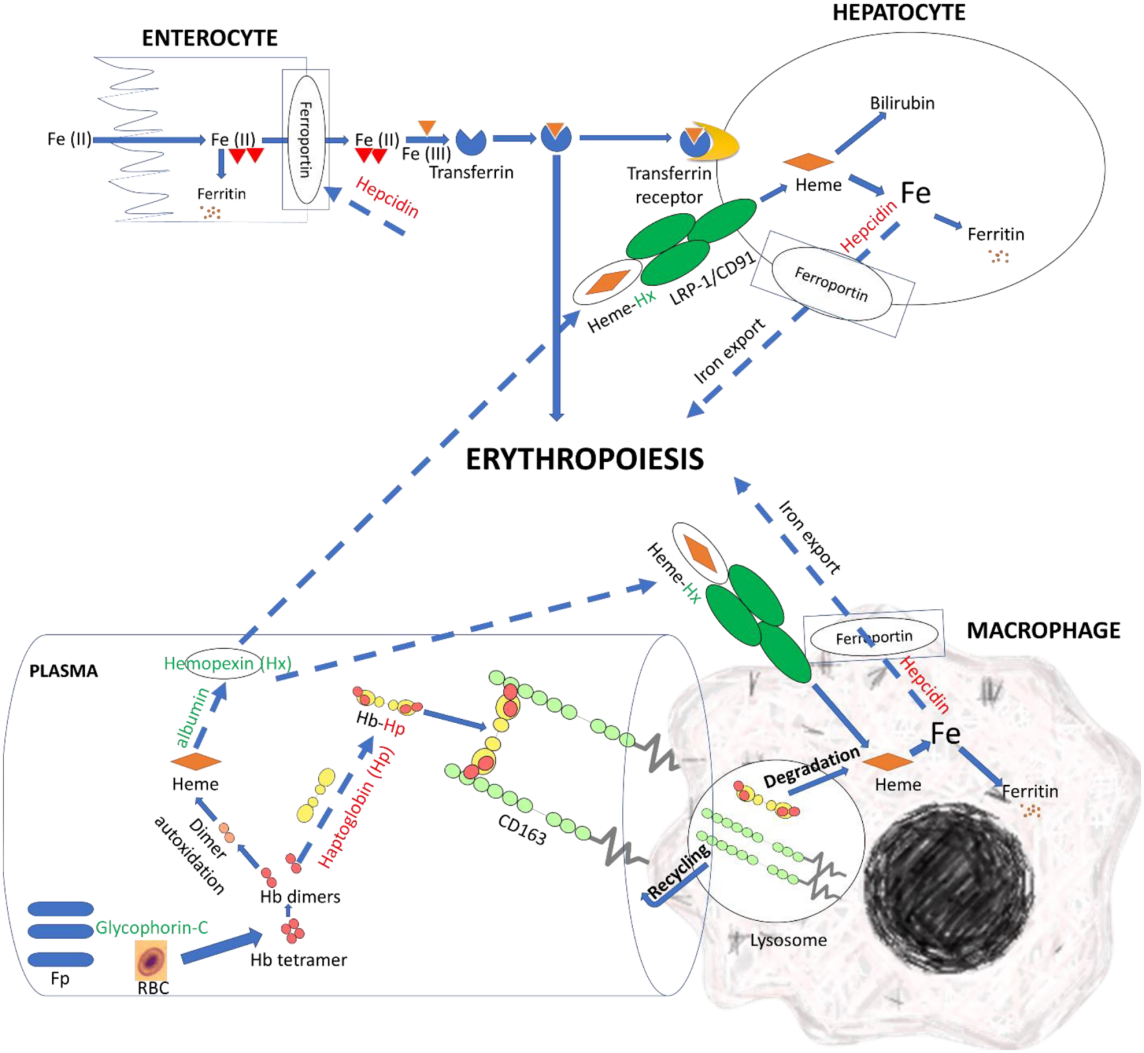
LncNATs complementary to hemolysis-related genes were DE in the resistant fish compared to susceptible fish on day 5 post-infection to promote clearance of free Hb and heme and enhance erythropoiesis. LncNATs of Hx, albumin, and glycophorin-C were upregulated in the resistant fish (green-coded), whereas lncNATs complementary to hepcidin and Hp were downregulated (red-coded). Glycophorin-C maintains the cell shape and membrane’s stability. Whereas Hp binds to Hb and transports it to the macrophages for catabolism. When Hp depletes, albumin transmits heme to Hx. The latter transports heme to the macrophage and hepatic cells, *via* receptor-mediated endocytosis, for catabolism. Low hepcidin expression allows iron export from macrophages and hepatocytes and iron import from enterocytes, promoting erythropoiesis. The proposed model suggests regulation of genes preventing pro-inflammatory effects of heme and reducing iron availability for bacterial growth in resistant fish. Dashed arrows indicate processes suggested in this study, whereas solid arrows refer to processes supported by information from the literature.

Efficient clearance of Hb and hemin can disrupt bacterial iron uptake and growth. Herein, we hypothesized that the resistant fish had a more efficient mechanism allowing clearance of free Hb and hemin **Figure 7**. We observed DE lncNATs and their complementary genes encoding specialized scavenger proteins that sequester Hb and hemin and transit them to the compartment where hemin can be metabolized by heme-oxygenases (upregulated in resistant fish) into less toxic metabolites (**Figure 6D** and **Supplementary File 2**). The lncNAT (OmyAS600043188) and its cognate protein-coding gene (hemopexin; Hx) were upregulated in the resistant fish after infection. Hx mainly sequesters heme and transports it to the hepatic cells, *via* receptor-mediated endocytosis, for catabolism and excretion (114).

Conversely, haptoglobin (Hp) and its complementary NAT were downregulated in the resistant fish on day 5. Hp is the primary hemoglobin-binding plasma protein that attenuates hemolytic disease’s pathophysiologic effects (114). Depletion of Hp occurs before the decline in Hx (114). Further, resistant fish had six upregulated lncNATs complementary to serum albumin. When Hp is depleted, albumin binds to heme to transport it to Hx, thus preventing the pro-inflammatory effects of heme and bacterial growth (114).

**Figure 8:**
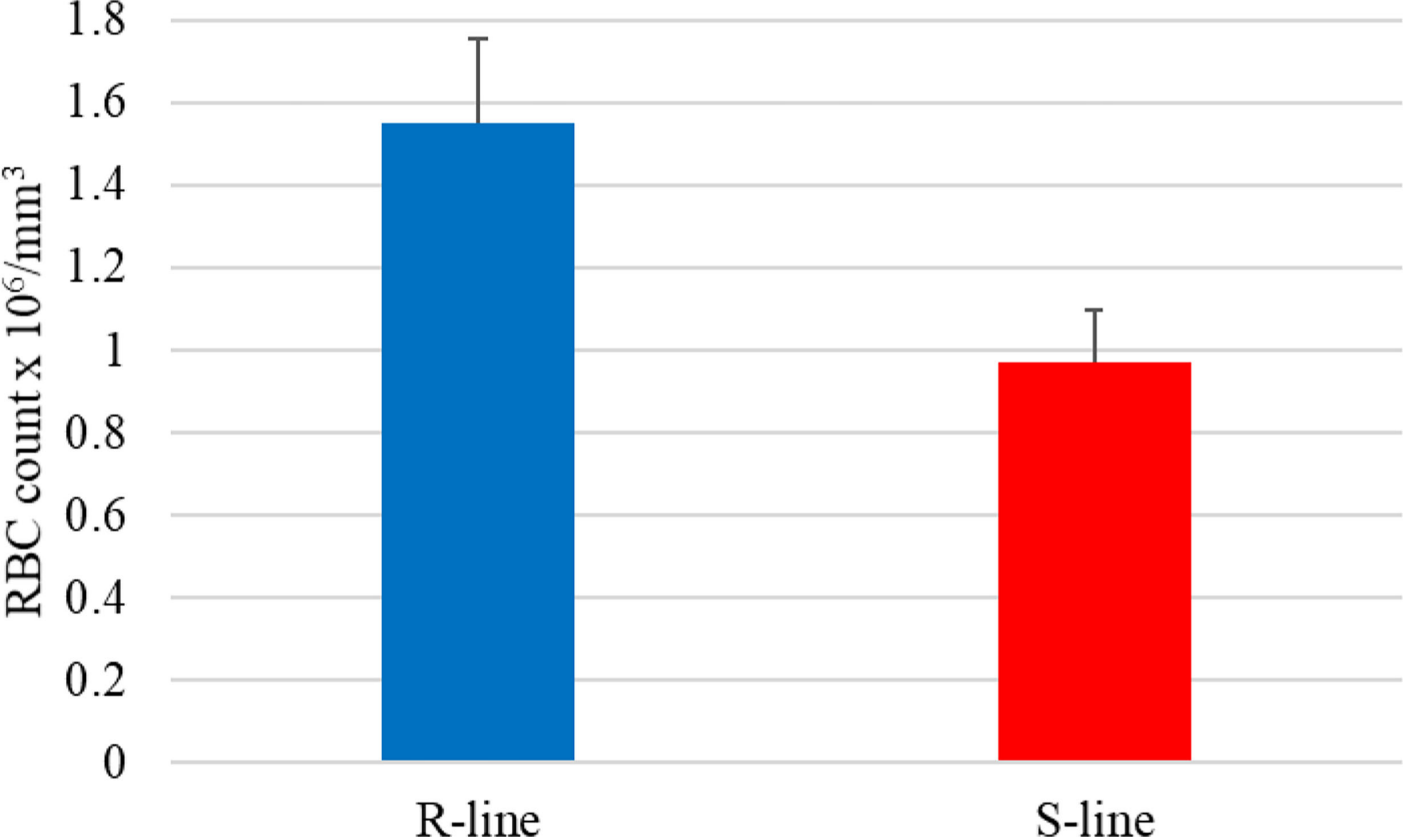
The average red blood cell count in the susceptible (S) genetic line was significantly lower than in fish from the resistant (R) genetic line (*p*-value < 0.02).

Notably, the NAT (OmyAS600022651) complementary to hepcidin (LOC100653444) was the most downregulated NAT in the resistant fish (**Table 2**). The two transcripts exhibited a high positive expression correlation across 24 RNA-Seq datasets (R = 0.85). Our recent study revealed the downregulation of two loci coding for hepcidin in resistant fish on day 5 post-infection (Fold change of -10.6 and -5.3) (115). Hepcidin is the master regulator of iron homeostasis. As a defense mechanism, hepcidin is induced to deplete and withhold extracellular iron from invading pathogens (116). Hepcidin binds to ferroportin, blocks transmembrane iron export from hepatocytes and macrophages (117), and inhibits iron absorption. Since hepcidin expression is suppressed by hypoxia and erythropoiesis (116, 118, 119), increased expression of hepcidin in susceptible fish could be associated with lower levels of erythropoiesis and hypoxia. Hypoxia has profound effects on promoting erythropoiesis (120) and reducing the development of bacterial infection and disease progression (121). Upregulation of hemoglobin subunits alpha and beta-1 following infection was previously reported in resistant fish (44, 67). Also, as shown in **Figure 8**, we noticed significantly higher red blood cell counts in the non-infected resistant than susceptible fish, suggesting a role for the red blood cells in disease resistance. It is worth mentioning that resistant fish had upregulated lipocalin (LOC110534089) on day 5. Lipocalin, secreted by neutrophils following infection, binds the bacterial siderophore and sequesters the siderophore–iron complex to prevent bacterial uptake (122). Mice lacking lipocalin exhibited high bacterial sensitivity (123). Altogether, these findings indicate that it is not only immune-related genes but also non-immune genes related to muscle contraction, proteolysis, and heme/iron metabolism that are suitable targets for future functional studies to identify causative genes/variants of susceptibility to the BCWD.

**Table 2:** Correlation between expression patterns of four DE lncNATs and their overlapping hepcidin genes across 24 RNA-Seq datasets.

DE lncNAT	Sense gene ID	R	log2FC	Padj	Comparison
OmyAS00022651	LOC100653444	0.85	5.05	2.27E-57	R_FP_1/R_FP_5
OmyAS00022651	LOC100653444	0.85	-1.79	5.67E-09	S_PBS_1/S_FP_1
OmyAS00022651	LOC100653444	0.85	-1.56	1.17E-04	R_PBS_1/R_Fp_1
OmyAS00022651	LOC100653444	0.85	3.96	2.70E-25	S_FP_5/R_FP_5
OmyAS00022649	LOC100653444	0.87	-2.04	4.00E-18	S_PBS_1/S_FP_1
OmyAS00022649	LOC100653444	0.87	-1.74	1.22E-16	S_PBS_5/S_FP_5
OmyAS00039423	LOC100135935	0.90	-5.19	5.93E-58	S_PBS_5/S_FP_5
OmyAS00039423	LOC100135935	0.90	-4.15	1.42E-80	S_PBS_1/S_FP_1
OmyAS00039424	LOC100135935	0.93	-5.54	9.80E-88	S_PBS_5/S_FP_5
OmyAS00039424	LOC100135935	0.93	-4.30	1.21E-64	S_PBS_1/S_FP_1
OmyAS00039424	LOC100135935	0.93	-1.17	1.02E-02	R_PBS_5/R_FP_5

OmyAS00022651 was the most differentially regulated lncNAT between the resistant and susceptible genetic line on day 5 post-infection (Padj < 0.05).

### DE lncNATs overlap with BCWD QTL reported in previous mapping studies

Notably, 94 DE lncNAT were located within 26 QTL regions previously identified in association with BCWD in rainbow trout. The QTL size ranges from ~622 kb up to 57.5 Mb. Most lncNATs overlapped with QTL on Omy3 (23 lncNATs; 24.5%), followed by Omy8 (14 lncNATs; 14.9%). The identified lncNATs have 72 cognate protein-coding genes. LncNATs complementary to hepcidin and myosin heavy chain were the top represented in the BCWD-associated QTL (**Table 3**). Three hepcidin complementary lncNATs are located in Omy2 QTL, and the other two lncNATs exist in Omy3 QTL. Five myosin heavy chain complementary lncNATs were found on four QTLs on Omy6, 11, 12, and 28. Furthermore, lncNATs complementary to proteolytic genes (chymotrypsin-like protease CTRL-1) and other genes essential for maintaining the erythrocyte shape and stability, such as glycophorin-C and protein 4.1 were identified on Omy3 QTL. All DE lncNATs overlapping with the previously identified BCWD QTL are provided in **Supplementary File 2**. Our results shed light, perhaps for the first time, on the potential role of iron homeostasis-, contraction-, and proteolysis-related genes in the disease progression in rainbow trout.

**Table 3:** DE lncNAT overlapping with previously published QTL for BCWD resistance in rainbow trout populations.

DE LncNAT	Sense gene	Annotation	log2FC	Comparison	Chr	QTL-start	QTL-end	Ref
OmyAS00022651	LOC100653444	Hepcidin	3.96	S_FP_5_R_FP_5	Omy2	20,000,000		(124)
OmyAS00022649	LOC100653444	Hepcidin	-2.04	S_PBS_1_S_FP_1	Omy2	20,000,000		(124)
OmyAS00022650	LOC100653444	Hepcidin	1.84	C_FP_5_S_FP_5	Omy2	20,000,000		(124)
OmyAS00038206	LOC110510788	chymotrypsin-like protease CTRL-1	2.04	S_FP_5_R_FP_5	Omy3	3,944,231	53,789,568	(125)
OmyAS00033057	LOC110520176	glycophorin-C	-1.34	S_FP_5_R_FP_5	Omy3	54,696,714		(126)
OmyAS00039424	LOC100135935	Hepcidin	-4.30	S_PBS_1_S_FP_1	Omy3	3,944,231	53,789,568	(125)
OmyAS00039423	LOC100135935	Hepcidin	-4.15	S_PBS_1_S_FP_1	Omy3	3,944,231	53,789,568	(125)
OmyAS00027826	c4	complement component 4	-1.80	S_PBS_5_S_FP_5	Omy3	3,944,231	53,789,568	(125)
OmyAS00027854	LOC110506699	protein 4.1	-1.01	S_PBS_1_S_PBS_5	Omy3	3,944,231	53,789,568	(125)
OmyAS00033059	LOC110520176	glycophorin-C	-1.18	S_PBS_5_R_PBS_5	Omy3	54,696,714		(126)
OmyAS00033058	LOC110520176	glycophorin-C	2.10	C_FP_5_S_FP_5	Omy3	54,696,714		(126)
OmyAS00027855	LOC110506699	protein 4.1	-1.04	C_PBS_5_C_FP_5	Omy3	3,944,231	53,789,568	(125)
OmyAS00078652	LOC110525076	myosin heavy chain, fast skeletal muscle	1.14	S_FP_5_R_FP_5	Omy6	4,355,841	15,516,680	(125)
OmyAS00078653	LOC110525076	myosin heavy chain, fast skeletal muscle	1.03	S_FP_5_R_FP_5	Omy6	4,355,841	15,516,680	(125)
OmyAS00094912	LOC100136615	differentially regulated trout protein 1	1.16	S_FP_5_R_FP_5	Omy7	15,368,940	15,991,566	(127)
OmyAS00094913	LOC100136615	differentially regulated trout protein 1	-1.63	S_PBS_1_S_FP_1	Omy7	15,368,940	15,991,566	(127)
OmyAS00097878	LOC110528266	engulfment and cell motility protein 2	-1.54	S_PBS_1_S_FP_1	Omy7	26,086,554	76,522,753	(125)
OmyAS00097879	LOC110528266	engulfment and cell motility protein 2	1.62	S_PBS_1_S_FP_1	Omy7	26,086,554	76,522,753	(125)
OmyAS00104412	LOC110530298	T-cell receptor alpha chain C region	1.06	R_FP_1_R_FP_5	Omy8	47,841,193	70,150,488	(125)
OmyAS00139685	LOC110535540	cAMP-responsive element modulator	1.31	S_FP_5_R_FP_5	Omy11	20,343,255	77,910,209	(125)
OmyAS00148282	LOC110536208	serum albumin 2	-1.44	S_FP_5_R_FP_5	Omy11	20,343,255	77,910,209	(125)
OmyAS00143441	LOC110536449	C-C motif chemokine 19	-1.04	S_PBS_5_S_FP_5	Omy11	20,343,255	77,910,209	(125)
OmyAS00148283	LOC110536208	serum albumin 2	-1.18	S_PBS_1_R_PBS_1	Omy11	20,343,255	77,910,209	(125)
OmyAS00143433	LOC110536447	myosin heavy chain, fast skeletal muscle	-1.48	R_PBS_1_R_PBS_5	Omy11	20,343,255	77,910,209	(125)
OmyAS00156027	LOC110538491	permeability factor 2	1.90	S_FP_5_R_FP_5	Omy12	18,722,763	76,000,009	(124)
OmyAS00156030	LOC110538492	permeability factor 2	2.07	S_FP_5_R_FP_5	Omy12	18,722,763	76,000,009	(124)
OmyAS00165042	LOC110538987	myosin heavy chain, fast skeletal muscle	-1.40	C_FP_1_R_FP_1	Omy12	68,915,818	78,021,920	(125)
OmyAS00325527	LOC110509125	myosin heavy chain, fast skeletal muscle	-1.56	R_PBS_1_R_PBS_5	Omy28	35,537,693	39,583,042	(124)

### Post-transcriptional effect of lncNAT expression (RNA-RNA duplexes)

LncNATs can affect all stages of gene expression, including transcriptional initiation and co-transcriptional and post-transcriptional processes (18). Many factors, including orientation, stability, cellular localization, and inherent features, can influence the lncNAT mechanism of action. Herein, we studied one of the possible mechanisms of action that can facilitate lncNATs to regulate protein-coding gene expression.

We studied the potential post-transcriptional effect of the antisense transcription. LncNATs can increase the stability of their target mRNAs by masking microRNA target sites, thus protecting mRNA from degradation (29). We identified 279 lncNATs that potentially interacted with sense coding loci. Out of them, 164 transcripts targeted the 3’UTR of sense mRNAs. Analysis of the microRNA target sites over their 3’UTRs revealed 2,916 binding sites for 172 microRNAs in the lncNAT-mRNA (3’UTR) duplex area. Of the 164 lncNATs, 110 were predicted to mask 2,151 microRNA binding sites (**Table 4** and **Supplementary File 3**). Most of these transcripts were DE in the infected ARS-Fp-S line on day 5 (n = 47).

**Table 4:** DE lncNATs interacting with the 3’UTR of cognate genes and masking microRNA target sites. ndG is the normalized binding free energy.

DE lncNATs	3’UTR Sense locus	Sense genes annotation	ndG	miRNA sites in the lncNAT-3’UTR interaction region
OmyAS00328473	leap-2b	liver-expressed antimicrobial peptide 2B	-618.63	mir-146d-3p, mir-146d-5p, mir-20a-5p, mir-17a-5p
OmyAS00307945	LOC110506924	selenoprotein S	-558.21	mir-20b-5p, mir-200b-5p, mir-21b-3p, mir-200b-3p, mir-20a-5p
OmyAS00039423	LOC100135935	Hepcidin	-415.12	mir-146a-5p
OmyAS00364414	socs3	suppressor of cytokine signaling 3	-282.48	mir-221-3p, mir-223-3p
OmyAS00075081	dpep1	dipeptidase 1	-235.36	mir-200c, mir-200b-5p
OmyAS00039424	LOC100135935	Hepcidin	-11.78	mir-146a-5p
OmyAS00005707	LOC100136344	serum albumin 1	-1.92	mir-125b-5p, mir-125c, mir-125a-5p
OmyAS00005706	LOC100136344	serum albumin 1	-1.92	mir-125b-5p, mir-125c, mir-125a-5p
OmyAS00202359	LOC110490451	Ig kappa-b4 chain C region	-1.15	mir-145a-5p, mir-2188-5p
OmyAS00239621	LOC110496824	immunoglobulin lambda-like polypeptide 5	-1.07	mir-125a-5p, mir-125b-5p, mir-200b-5p, mir-200c
OmyAS00038206	LOC110510788	chymotrypsin-like protease CTRL-1	-0.95	mir-206-5p
OmyAS00056563	lyz2	lysozyme II	-0.93	let-7d-3p, mir-196a-5p, mir-196b-5p, mir-184a, let-7a-5p
OmyAS00156030	LOC110538492	permeability factor 2	-0.87	mir-21b-3p, mir-212a-3p
OmyAS00033058	LOC110520176	glycophorin-C	-0.66	mir-212a-5p, mir-212b-3p
OmyAS00144899	LOC100135878	chemokine CK-1 precursor	-0.63	mir-143-5p
OmyAS00074066	LOC100136024	interleukin-1-beta	-0.62	mir-138a
OmyAS00143441	LOC110536449	C-C motif chemokine 19	-0.56	mir-146d-3p, mir-146d-5p, mir-212a-5p, mir-212b-3p
OmyAS00075100	LOC100135921	Haptoglobin	-0.25	mir-122-5p, mir-19b

There were binding sites to microRNAs known to have a role in bacterial infection. These miRNAs include mir-146, mir-125, mir-132, mir-20, mir-223, mir-21, mir-212, mir-200, and mir-17 (128). The presence of microRNA binding sites in the 3’UTR of immune-relevant genes suggests a role for lncNAT-mRNA duplex in regulating the immune response. For example, C-C motif chemokine 19 has binding sites to mir-146d-3p, mir-146d-5p, mir-212a-5p, and mir-212b-3p. IL-6R alpha precursor has a binding site to mir-146a-5p, whereas suppressor of cytokine signaling 3 (SOCS3) has binding sites to mir-223 and mir-221. MiR-146a has an important role in controlling the proliferation of immune cells and suppressing inflammatory responses (129), whereas miR-221 negatively regulates SOCS3 (130). Further, mannan-binding lectin serine peptidase 2, which plays a crucial role in the activation of complement system, has binding sites to mir-132a, mir-132b, mir-146a-5p, mir-200b-5p, mir-212a-5p, mir-212b-3p, and mir-21a-5p.

It is worth mentioning that most of the DE lncNATs complementary to genes involved in the cellular iron/heme homeostasis and proteolysis mask miRNA binding sites in the 3’UTR of mRNA transcribed from the opposite strand. These include lncNATs complementary to hepcidin, haptoglobin, serum albumin 1, selenoprotein S, aminopeptidase N, carboxypeptidase B, dipeptidase 1, and chymotrypsin-like protease CTRL-1. In humans, the β-site APP-cleaving enzyme 1 gene (BACE1) forms an RNA-RNA duplex with the antisense transcript. The duplex area masks the binding site of miR 485 5p and augments the translation of BACE1 (29). Also, antisense transcription modulates IFN-α1 mRNA stability by masking the miR-1270 binding site (131). Taken together, this study suggests that lncNATs are involved in regulating gene expression and driving disease progression. Detailed information about all possible lncNAT-mRNA duplex and miRNA binding sites is listed in **Supplementary File 3**.

## Conclusions

We identified thousands of antisense transcriptions from the complementary strands to protein-coding genes in the rainbow trout reference genome. We investigated the potential role of these antisense transcripts in shaping host-pathogen interactions in selectively bred, resistant-, control-, and susceptible-line rainbow trout following a challenge with *Fp*. The study identified DE antisense transcripts overlapping and exhibiting expression correlation with genes coding for immune-, proteolysis-, and hemolysis-related proteins. The hemolysis-related genes included Hx, Hp, hepcidin, complement factor H, and albumin. LncNATs complementary to hepcidin, a master regulator of iron homeostasis, were exceptionally upregulated in susceptible fish on day 5 post-infection. About 16% of DE lncNATs were located in 26 QTL regions previously identified in association with BCWD in rainbow trout. Many antisense transcripts showed positive expression correlation with their sense counterpart genes. A possible mechanism that may explain the synchronized expression is the ability of antisense transcripts to form RNA-RNA duplexes with their cognate protein-coding genes, increasing their stability by masking miRNA binding sites. This study improves our understanding of fish resistance to Fp infection and provides suitable targets for future functional genomics studies.

## Data availability statement

The original contributions presented in the study are included in the article/**Supplementary Material**. Further inquiries can be directed to the corresponding author.

## Ethics statement

The animal study was reviewed and approved by The NCCCWA Institutional Animal Care and Use Committee Protocols #053 and #076. Blood samples used in this study were obtained from the USDA/NCCCWA (Dr. Gregory D. Wiens).

## Author contributions

MS and AA conceived and designed the study; AA and MS analyzed the data and wrote the paper. All authors contributed to the article and approved the submitted version.
